# Does surgical plethysmographic index-guided analgesia affect opioid requirement and extubation time? A systematic review and meta-analysis

**DOI:** 10.1007/s00540-022-03094-z

**Published:** 2022-08-20

**Authors:** Shao-Chi Hung, Wei-Ti Hsu, Chi-Lin Fu, Yu-Wen Lai, Mei-Ling Shen, Kuen-Bao Chen

**Affiliations:** 1 Department of Anesthesiology, China Medical University Hospital, No. 2, Yude Rd., North Dist., Taichung City, 404332 Taiwan ROC; 2 Graduate Institute of Biomedical Sciences, College of Medicine, China Medical University, Taichung, Taiwan ROC; 3 Department of Nursing, China Medical University Hospital, Taichung, Taiwan ROC; 4 School of Nursing, China Medical University, Taichung, Taiwan ROC; 5 Department of Anesthesiology, Taichung Tzu-Chi Hospital, Taichung, Taiwan ROC; 6 Department of Anesthesiology, College of Medicine, China Medical University, Taichung, Taiwan ROC

**Keywords:** Surgical plethysmographic index, Analgesia, Opioid requirement, Extubation time, Hypnosis monitor, Enhanced recovery after surgery

## Abstract

**Purpose:**

This meta-analysis of all relevant clinical trials investigated surgical plethysmographic index (SPI)-guided analgesia’s efficacy under general anesthesia for perioperative opioid requirement and emergence time after anesthesia.

**Methods:**

PubMed, Embase, Web of Science, and Cochrane Library were searched up to January 2022 to identify clinical trials comparing SPI-guided and conventional clinical practice for patients who underwent general anesthesia. With the random-effects model, we compared intraoperative opioid consumption, emergence time, postoperative pain, analgesia requirement, and incidence of postoperative nausea and vomiting (PONV).

**Results:**

Thirteen randomized controlled trials (RCTs) (*n* = 1314) met our selection criteria. The overall pooled effect sizes of all RCTs indicated that SPI-guided analgesia could not significantly reduce opioid consumption during general anesthesia. SPI-guided analgesia accompanied with hypnosis monitoring could decrease intraoperative opioid consumption (standardized mean difference [SMD] − 0.31, 95% confidence interval [CI] − 0.63 to 0.00) more effectively than SPI without hypnosis monitoring (SMD 1.03, 95% CI 0.53–1.53), showing a significant difference (*p* < 0.001). SPI-guided analgesia could significantly shorten the emergence time, whether assessed by extubation time (SMD − 0.36, 95% CI − 0.70 to − 0.03, *p* < 0.05, *I*^2^ = 67%) or eye-opening time (SMD − 0.40, 95% CI − 0.63 to − 0.18, *p* < 0.001, *I*^2^ = 54%). SPI-guided analgesia did not affect the incidence of PONV, postoperative pain, and analgesia management.

**Conclusion:**

SPI-guided analgesia under general anesthesia could enhance recovery after surgery without increasing the postoperative complication risk. However, it did not affect intraoperative opioid requirement. Notably, SPI-guided analgesia with hypnosis monitoring could effectively reduce intraoperative opioid requirement.

## Introduction

Recently, owing to the impact of Enhanced Recovery After Surgery (ERAS), which aimed to improve patient outcomes and accelerate recovery [1], the prevailing trend of anesthesia focused on decreasing opioid use in pain management to prevent postoperative complications [2, 3]. Intraoperative opioids could provide sufficient analgesia and reduce cardiovascular dynamic fluctuations. However, opioid overdose could likely result in postoperative respiratory depression, nausea and vomiting, and ileus [4–7]. Thus, general anesthesia with opioid sparing is associated with rapid awakening, early mobilization, and reduced number of hospital days [8, 9].

Nowadays, various monitoring techniques provide quantification of autonomic activation or nociception–antinociception balance following noxious stimuli and are applied to opioid guidance during general anesthesia. The most monitoring techniques are based on the heart rate-derived variables, plethysmographic pulse wave amplitude, pupillometry, and muscle activity [9, 10]. The management of intraoperative analgesia administration with nociception–antinociception balance monitor helps avoid opioid overdose and hemodynamic fluctuations and shortens the emergence time after anesthesia [11, 12].

The surgical plethysmographic index (SPI) (GE Healthcare, Helsinki, Finland), also known as the surgical stress index, is a normalized score calculated using the combination of normalized heart beat interval (HBI_norm_) and plethysmographic pulse wave amplitude (PPGA_norm_) (SPI = 100 – [0.7 × PPGA_norm_ + 0.3 × HBI_norm_]). The SPI ranges from 0 to 100, with higher values indicating greater stress responses. Meanwhile, an SPI represents the mean stress level during anesthesia. The SPI can be used to measure the sympathetic activity of the autonomic nervous system and is highly correlated with nociceptive stimulus intensity and opioid concentration [10, 13, 14]. SPI is more favorable in clinical practice because of several advantages; it requires only finger pulse oximetry without additional medical consumables to provide noninvasive and continuous monitoring and has a better effect on intraoperative opioid sparing than that of nociception monitors [11, 15].

Previous several studies had reviewed articles published before 2018, which focused on the effect of nociception monitor-guided analgesia during general anesthesia [15–18]. Yang et al. and Meijer et al. reviewed the effects of different nociception monitors on guided opioid administration. However, they did not analyze the postoperative emergence time [15, 18]. Gruenewald et al. and Won et al. reviewed clinical trials on the effect of SPI on intraoperative opioid requirement, emergence time, and perioperative adverse events [16, 17]. However, the meta-analysis results are limited or uncertain. Furthermore, the relevant clinical trials of the abovementioned reviews were published before 2018. Moreover, several recently published related clinical trials [19–22] have shown conflicting results about intraoperative opioid consumption. To explore the possible reasons for the difference and efficacy of accelerating recovery after surgery, we conducted this meta-analysis with all relevant clinical trials; thus, far to clarify the efficacy of SPI-guided analgesia under general anesthesia for intraoperative opioid consumption, emergence time, postoperative nausea, vomiting (PONV), and postoperative pain.

## Methods

Three authors independently (Hung, Shen, and Fu) used four common electronic databases, namely, PubMed, Embase, Web of Science, and Cochrane Library, to search clinical trials about the SPI-guided analgesia for perioperative opioid requirement from the inception date to January 2022. Relevant review articles were also searched for further studies. The following keywords were used to search for potential literature from the databases: “surgical pleth index,” “opioid,” and “general anesthesia.” The search strings of PubMed were (general anesthesia) AND (analgesia OR opioid) AND ([surgical pleth index] OR [surgical stress index] OR [plethysmography] OR [Photoplethysmography]). Three independent authors (Hung, Hsu, and Shen) assessed the title, abstract, and full text of the all identified articles for eligibility with the following inclusion criteria: (1) randomized controlled trials (RCTs) in humans, (2) participants underwent elective surgery by general anesthesia with intubation, (3) the intervention group used SPI-guided analgesia administration, and (4) the outcome assessment included intraoperative opioid consumption. The exclusion criteria were as follows: (1) review articles, protocols, conference papers, case reports, letters, editorials; (2) no intubation under general anesthesia; (3) control group using any kind of nociception monitor-guided analgesia (i.e., SPI, pupillary pain index, and nociception level). Three authors (Hung, Fu, and Lai) independently assessed the quality of all included trials according to the Cochrane Collaboration’s tool for assessing the risk of bias [23]. The judgment results of each item were divided into “low risk,” “unclear risk,” or “high risk” based on the relevant data from the included studies. In the case of disagreements in judgment results, the advising professor (Chen) joined the discussions to resolve conflicts.

### Data extraction

The primary outcomes were comparisons of intraoperative opioid consumption and emergence time between SPI-guided analgesia and conventional clinical practice. The secondary outcomes were the incidence of PONV, postoperative pain, and analgesia requirement in the postanesthesia care unit (PACU). Three authors independently (Hung, Shen, and Lai) extracted the clinical information and data from all included studies. When public data were incomplete, we would email authors to obtain missing data. On account that all analysis data were extracted from public literature, the institutional review board review was not required.

### Statistical analysis

As different units and different pain scales were used between studies, the effect sizes (ESs) for the continuous outcomes such as opioid consumption, emergence time, pain scores, and analgesia requirements were calculated in SMDs with 95% CI. The ESs for the dichotomous outcomes, such as PONV, were calculated in risk ratios. A *p* value of < 0.05 was considered statistically significant for the analysis of ESs. The random-effects model was used to estimate the pooled ESs. Heterogeneity was assessed by *I*-square (*I*^2^) statistics, and an *I*^2^ of more than 50% was considered to indicate substantial heterogeneity. Publication bias was investigated using a funnel plot [24], and visual observation of funnel plot symmetry was employed to assess the presence of potential publication bias. Subgroup meta-analysis was conducted to investigate intraoperative opioid consumption with or without hypnosis monitors. Furthermore, we performed a subgroup meta-analysis to investigate whether the differences in the study protocol affected the difference in the intraoperative opioid consumption between the SPI-guided group and the control group. The Review Manager 5 software (version 5.4, The Nordic Cochrane Centre, Copenhagen, Denmark) was used to process all meta-analyses.

## Results

**Fig. 1:**
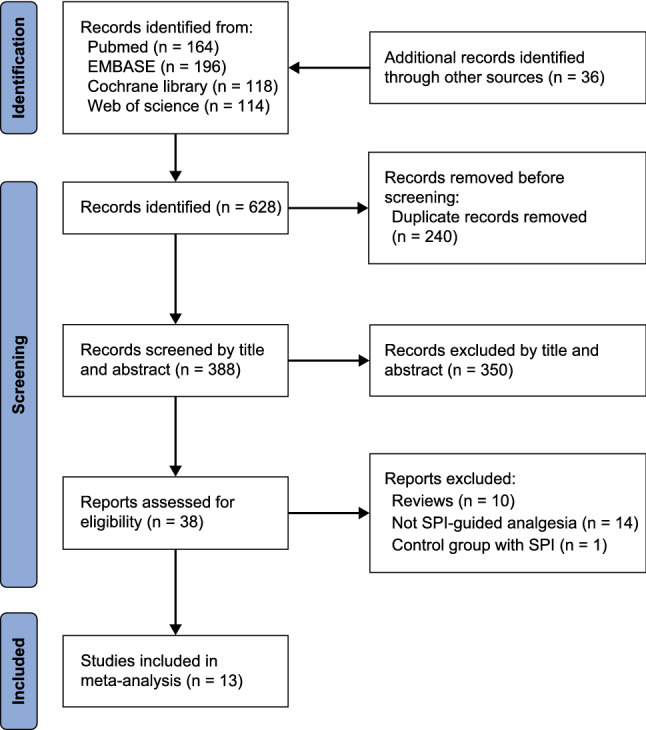
PRISMA flow diagram of literature search and study selection

Initially, 628 articles were obtained from four databases and relevant review articles. After excluding 240 articles because of duplication and 375 articles for not meeting the selection criteria, 13 studies were included in the meta-analysis (Fig. 1).

The characteristics of the included studies are presented in Table 1. Thirteen RCTs were included, with a total of 1314 participants, in our meta-analysis. These studies were published between 2010 and 2021. The age of the participants ranged from 3 to 74 years. They had an American Society of Anesthesiologists physical status classification of I–III, and underwent elective surgery including orthopedic surgery [25–27], ear–nose–throat surgery [11, 27, 28], laparoscopic cholecystectomy [19, 21, 29, 30], radical retropubic prostatectomy [20, 22], gynecological surgery [26, 27], thyroidectomy [12], adenotonsillectomy [31], maxillofacial, and trauma surgery [27].

**Table 1:** Summary of general characteristics of the included studies

Authors	Journal	Participants	Age (years)	ASA	Types of surgery	Hypnotic/opioid	Hypnosis monitor	Outcome assessments
Chen et al. [11]	Anesthesiology	SPI: 40 Control: 40	SPI: 47 ± 17 Control: 46 ± 17	I ~ II	Elective ear/nose/throat surgery	Propopol/remifentanil	BIS	Eye-open time, PONV, VAS
Bergmann et al. [25]	British Journal of Anesthesia	SPI: 76 Control: 75	SPI: 48 (18–69)^a^ Control: 44 (18–74)	I ~ III	Outpatient orthopaedic surgery	Propopol/remifentanil	Entropy	Extubation time, PONV, NRS, eye-open time
Gruenewald et al. [26]	British Journal of Anesthesia	SPI: 42 Control: 40	SPI: 37 (33–40)^b^ Control: 41 (37–45)	I ~ II	Elective surgery (gynecological and orthopedic procedures)	Sevoflurane/sufentanil	BIS	Extubation time, NRS, PACU analgesia
Colombo et al. [29]	Minerva Anestesiol	SPI: 30 Control: 30	SPI: 46.6 ± 12.2 Control: 49.9 ± 11.4	I ~ II	Elective laparoscopic cholecystectomy	Propopol/remifentanil	Entropy	NRS
Park et al. [31]	Anesthesiology	SPI: 21 Control: 24	SPI: 7 (5–10)^a^ Control: 7 (3–10)	I	Elective adenotonsillectomy	Sevoflurane/fentanyl	Entropy	Extubation time, PONV, eye-open time, CHEOPS, PACU analgesia
Won et al. [12]	Medicine	SPI: 23 Control: 22	SPI: 54 (26–65)^a^ Control: 42 (25–61)	I ~ II	Elective thyroidectomy	Sevoflurane/oxycodone	BIS	Extubation time, PONV, NRS, PACU analgesia
Jain et al. [19]	Minerva Anestesiologica	SPI: 68 Control: 65	SPI: 38.4 ± 12.0 Control: 40.3 ± 12.3	I ~ II	Laparoscopic cholecystectomy	Sevoflurane/fentanyl	BIS	Eye-open time, PONV, VAS, PACU analgesia
Funcke et al. [20]	Anesth Analg	SPI: 12 Control: 12	SPI: 64 ± 7 Control: 61 ± 6	II ~ III	Radical retropubic prostatectomy	Sevoflurane/sufentanil	N/A	Extubation time, NRS, PACU analgesia
Kim et al. [21]	Anesth Analg	SPI: 20 Control: 10	SPI: 46.6 ± 10.3 Control: 50.2 ± 7.8	I ~ II	Laparoscopic cholecystectomy	Propopol/remifentanil	Entropy	Extubation time, NRS, eye-open time
Funcke et al. [22]	British Journal of Anesthesia	SPI: 23 Control: 24	SPI: 62 ± 7 Control: 63 ± 10	II ~ III	Radical retropubic prostatectomy	Propopol/remifentanil	N/A	Extubation time, PONV, NRS, PACU analgesia
Gruenewald et al. [27]	European Journal of Anesthesiology	SPI: 246 Control: 248	SPI: 48 ± 15 Control: 48 ± 16	N/A	Gynecological, ear/nose/throat and Maxillofacia, orthopedic, trauma	Propopol/remifentanil	Entropy	Eye-open time, PONV, VAS
Guo et al. [30]	BMC Anesthesiol	SPI: 31 Control: 31	SPI: 47.1 ± 11.6 Control: 48.8 ± 13.4	I ~ II	Laparoscopic cholecystectomy	Propopol/fentanyl	BIS	Extubation time, PONV, VAS
Stasiowski et al. [28]	Journal of Clinical Medicine	SPI: 31 Control: 30	SPI: 47.7 ± 13.9 Control: 49 ± 15.4	I ~ III	Endoscopic sinus surgery	Propopol/remifentanil	Entropy	BBS

The data are represented as number of participants; mean ± SD

*ASA* American Society of Anesthesiologists physical status classification, *SPI* Surgical Pleth Index, *BIS* Bispectral Index, *PONV* Postoperative nausea and vomiting, *VAS* Visual Analogue Scale, *NRS* Numerical rating scale, *CHEOPS* Children’s Hospital of Eastern Ontario Pain Scale, *PACU* Postanesthesia care unit, *BBS* Boezaart bleeding scale, *N/A* Not available

^a^Median (range)

^b^Mean (95% CI)

General anesthesia was maintained by using inhalation anesthesia with sevoflurane in five studies [12, 19, 20, 26, 31] or by intravenous anesthesia with propofol in eight studies [11, 21, 22, 25, 27–30]. Intraoperative analgesia was administered with continuous remifentanil infusion in seven studies [11, 21, 22, 25, 27–29], intravenous anesthesia with fentanyl in three studies [19, 30, 31], sufentanil in two studies [20, 26], or oxycodone in one study [12]. Participants received hypnosis monitoring with bispectral index (BIS) in five studies [11, 12, 19, 26, 30] or entropy in six studies [21, 25, 27–29, 31], or did not receive hypnosis monitoring in two studies [20, 22].

Emergence time after anesthesia was reported with extubation time in eight studies [12, 20–22, 25, 26, 30, 31] or eye-opening time in six studies [11, 19, 21, 25, 27, 31]. Postoperative pain was measured by using the numerical rating scale in seven studies [12, 20–22, 25, 26, 29], visual analog scale in four studies [11, 19, 27, 30], or Children’s Hospital of Eastern Ontario Pain Scale in one study [31]. PONV was reported in eight studies [11, 12, 19, 22, 25, 27, 30, 31], and six studies reported the requirements of analgesia in the PACU [12, 19, 20, 22, 26, 31].

**Fig. 2:**
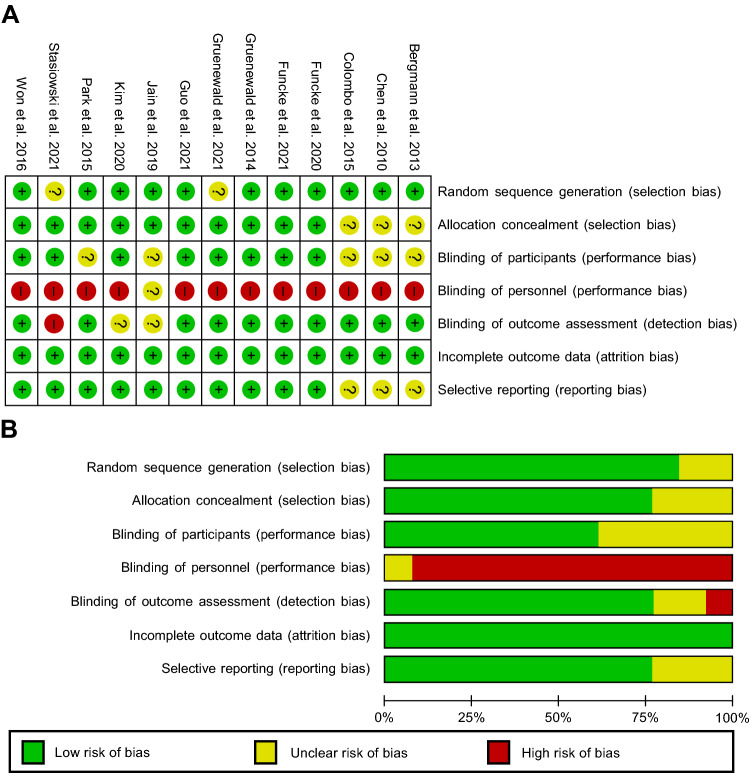
Quality assessment **A** risk of bias summary and **B** risk of bias graph across all included studies. Most trials had a low risk of bias, and the blinding of personnel was associated with a high risk of bias

The results of the risk of bias assessment showed that the majority of the trials had a low risk of bias, except for one of the performance bias (Fig. 2). Almost all studies had a high risk of bias for the blinding of the personnel because the attending anesthesiologists were responsible for administering opioids based on SPI or vital signs.

**Fig. 3:**
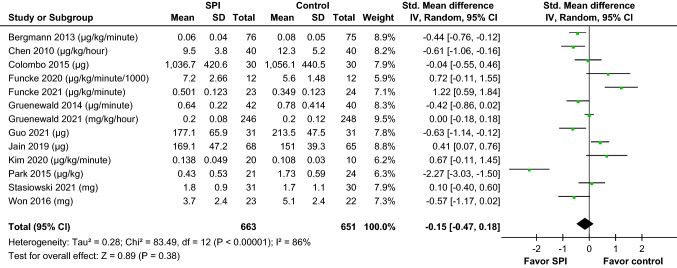
Forrest plots indicated the pooled effect sizes of SPI-guided analgesia compared with the control on intraoperative opioid consumption. The SPI-guided analgesia had no significant effect on reducing intraoperative opioid consumption. *SPI* surgical plethysmographic index

**Fig. 4:**
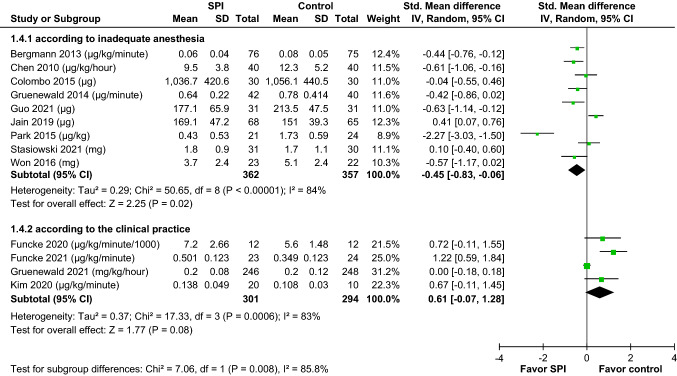
Forrest plots were used in the subgroup analysis of the effect of SPI-guided analgesia compared with the control on intraoperative opioid consumption, according to different study designs in the control groups (inadequate anesthesia vs. individual experience practice). The use of SPI-guided analgesia had a superior effect on decreasing intraoperative opioid consumption when preventing the effect of the individual experience of anesthesiologists in the control group. *SPI* surgical plethysmographic index

In the meta-analysis of all 13 studies, the results revealed that no difference in opioid consumption between the SPI-guided analgesia group and the control group (SMD =  − 0.15, 95% CI − 0.47 to 0.18, *p* = 0.38). Meanwhile, significant heterogeneity was noted (*I*^2^ = 86%,* p* for *I*^2^ < 0.001) (Fig. 3). When we reviewed the literature, the opioid administration was adjusted according to the SPI-guided value in the SPI groups of all studies. The anesthesiologists increased the opioid dosage if the SPI was > 50 and if it persisted for > 15 s. The attending anesthesiologists adjusted the opioid dosage to maintain the SPI value within a defined range. However, regarding the control group, in nine studies [11, 12, 19, 25, 26, 28–31], opioid administration was adjusted carefully according to “inadequate anesthesia,” which was defined as either heart rate (HR) or mean arterial pressure (MAP) increasing > 20% above the baseline. In the other four studies [20–22, 27], opioid administration was adjusted according to the individual clinical practice by the anesthesiologist without any standard guidelines. Thus, subgroup analyses by study designs were conducted. The result of the subgroup analysis showed that the SPI group significantly had decreased opioid consumption (SMD − 0.45, 95% CI − 0.83 to − 0.06, *p* = 0.02) when the opioid administration in the control group was adjusted according to “inadequate anesthesia.” Opioid consumption was slightly different between the two groups, but an increasing trend was noted in the SPI-guided group (SMD 0.61, 95% CI − 0.07 to 1.28, *p* = 0.08) when opioid administration in the control group was adjusted according to the individual clinical practice by the anesthesiologist. The difference between these subgroups was significant (*p* < 0.05) (Fig. 4). The subgroup analysis showed that the use of SPI-guided analgesia had a superior effect of decreasing intraoperative opioid consumption when avoiding individual experience practice of anesthesiologists in the control groups.

**Fig. 5:**
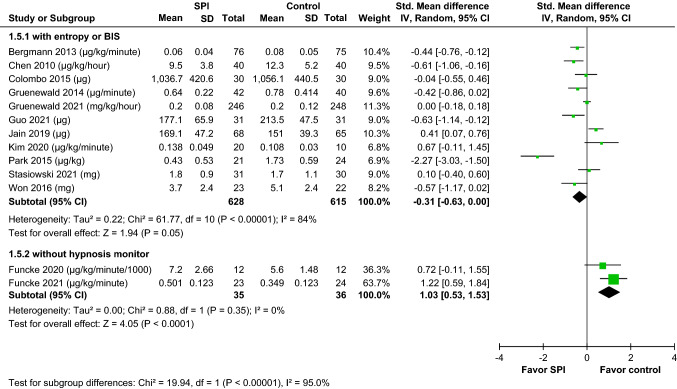
Forrest plots were used in the subgroup analysis of the effect of SPI-guided analgesia compared with the control on intraoperative opioid consumption with or without hypnosis monitoring (with entropy or BIS vs. without hypnosis monitoring). The use of SPI-guided analgesia with hypnosis monitoring had a superior effect on decreasing intraoperative opioid consumption. *BIS* bispectral index, *SPI* surgical plethysmographic index

**Fig. 6:**
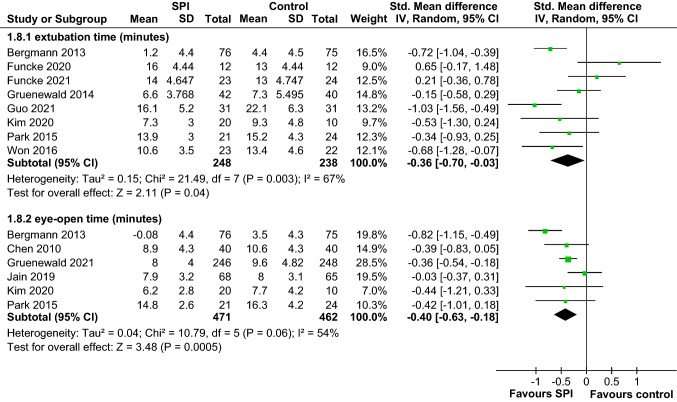
Forrest plots were used to analyze the pooled effect sizes of SPI-guided analgesia compared with the control on extubation time (**A**) and eye-opening time (**B**). The SPI-guided analgesia had a significant effect on reducing extubation time and eye-opening time. *SPI* surgical plethysmographic index

Furthermore, we conducted subgroup analysis by the use or nonuse of hypnosis monitoring. Participants received monitoring of entropy or BIS during general anesthesia in 11 studies [11, 12, 19, 21, 25–31]. The result of the subgroup analysis showed that the opioid consumption was slightly different between the two groups, but a decreasing trend was found in the SPI group (SMD − 0.31, 95% CI − 0.63 to 0.00, *p* = 0.05) when using entropy or BIS under general anesthesia. In the other two studies [20, 22], participants did not receive any hypnosis monitoring during general anesthesia. The result of the subgroup analysis revealed the SPI-guided group significantly had increased opioid consumption (SMD 1.03, 95% CI 0.53–1.53,* p* < 0.001). The difference between these subgroups was significant (*p* < 0.001) (Fig. 5). The subgroup analysis also demonstrated that the use of SPI-guided analgesia with hypnosis monitoring had a superior effect of decreasing intraoperative opioid consumption. The emergence time was assessed by the extubation time, which was defined as the period between the end of surgery to the removal of the tracheal tube, or by the eye-opening time, which was defined as the interval from the end of surgery to eye-opening on command. As presented in Fig. 6, SPI-guided analgesia had a significant effect on reducing the extubation time (SMD − 0.36, 95% CI − 0.70 to − 0.03, *p* < 0.05, *I*^2^ = 67%). Meanwhile, the use SPI could shorten the eye-opening time (SMD − 0.40, 95% CI − 0.63 to − 0.18, *p* < 0.001, *I*^2^ = 54%).

**Fig. 7:**
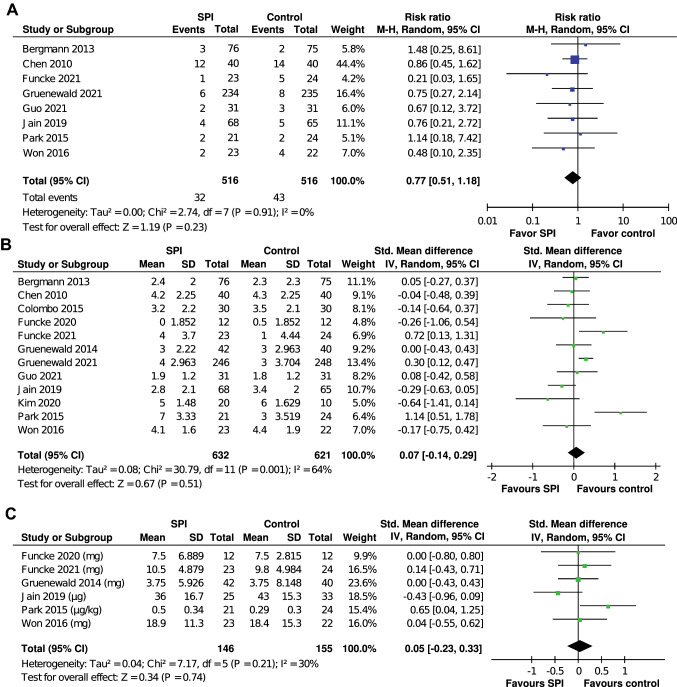
Forrest plots indicated the pooled effect sizes of SPI-guided analgesia and control on postoperative nausea and vomiting (**A**), postoperative pain scores (**B**), and analgesic requirement among patients in the PACU (**C**). SPI-guided analgesia had no significant effect on the incidence of PONV, degree of pain, or amount of analgesics among patients in the PACU. *PACU* postanesthesia care unit, *SPI* surgical plethysmographic index

As illustrated in Fig. 7, no difference was found between the SPI-guided group and the control group regarding PONV risk (SMD = 0.77, 95% CI 0.51–1.18, *p* = 0.23, *I*^2^ = 0%), pain scores (SMD = 0.07, 95% CI − 0.14 to 0.29, *p* = 0.51, *I*^2^ = 64%), and analgesic requirement (SMD = 0.05, 95% CI − 0.23 to 0.33, *p* = 0.74, *I*^2^ = 30%) in the PACU. Those results indicated that the use of SPI-guided analgesia did not particularly increase or decrease the incidence of PONV, degree of pain, or amount of analgesics in the PACU.

**Fig. 8:**
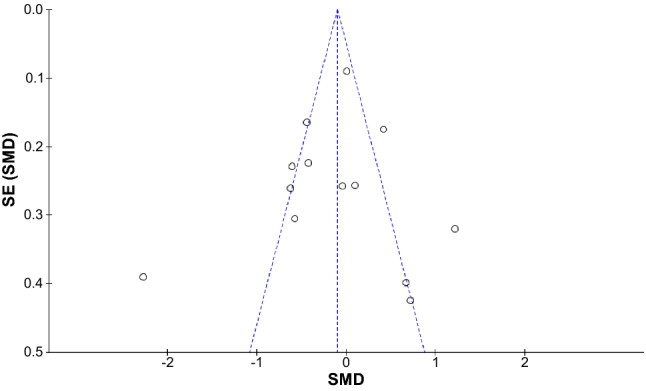
Funnel plot of the 13 trials based on opioid consumption to explore publication bias. The standardized mean difference (SMD) is on the *X* axis and the standard error on the *Y* axis

The funnel plot of the 13 trials based on opioid consumption did not indicate evidence of a publication bias, as it demonstrated a symmetrical shape on visual inspection (Fig. 8).

## Discussion

This study focused on whether the use of SPI to guide opioid administration reduced opioid requirement and effectively accelerated recovery after surgery, in line with ERAS. In comparison with the number of RCTs included in the previous meta-analyses [15–18], we included seven more RCTs [19–22, 27, 28, 30]. Our meta-analysis, including 13 RCTs, revealed no significant decrease in intraoperative opioid consumption, but effectively demonstrated shortened extubation and eye-opening times in the SPI-guided group. Moreover, significant heterogeneity was noted in the preliminary results. However, the quality assessment of the included studies revealed a high risk of performance bias; thus, we conducted multiple subgroup analyses of the study designs to explore possible underlying factors affecting intraoperative opioid requirement.

Our subgroup analysis revealed a significantly greater reduction in intraoperative opioid consumption in the SPI-guided analgesia group than that in the control group, in which opioid administration was adjusted based on the “inadequate anesthesia” rather than the anesthesiologist’s personal experience (Fig. 4). Recently, opioid-free perioperative care strategies and the impact of ERAS have gained attention. Based on this medical concept, attending anesthesiologists may reduce intraoperative opioid use during surgery [32, 33]. Pain management based on the individual experience practice of anesthesiologists might underestimate or ignore the development of painful stimuli, which could result in the early use of antihypertensive drugs to suppress cardiovascular changes. Hence, the performance bias caused by individual anesthesiologists should be eliminated using the standard normative research steps, which include inadequate anesthesia, defined as an abnormal range of HR or MAP [34]. However, pain management based on inadequate anesthesia might overestimate pain stimuli due to hemodynamic instability, which can lead to the overuse of opioids and delayed intervention of antihypertensive drugs. Therefore, SPI-guided analgesia could appropriately manage pain stimuli, reduce the application of unnecessary opioids, and properly indicate the use of antihypertensive drugs to control hemodynamic changes.

Another subgroup analysis in 11 studies with hypnosis monitoring such as entropy or BIS showed that the use of SPI-guided analgesia had a superior effect of decreasing intraoperative opioid consumption (Fig. 5). BIS-guided general anesthesia could reduce the requirement for propofol or volatile anesthetics [32, 33]. Keeping BIS values within the acceptable range (40–60) was related to a more pronounced fluctuation in cardiovascular dynamics and required increasing analgesic consumption to maintain stable hemodynamics [34]. SPI could reduce the unnecessary use of opioids for nonpainful stimuli. Hemodynamic instability due to nonpainful stimuli should be treated with vasodilators or vasopressors. Consequently, SPI accompanied with hypnosis monitoring can provide better effects on opioid administration during general anesthesia.

This meta-analysis further showed that the extubation time or eye-opening time was significantly shorter during general anesthesia with SPI than without it. Postoperative respiratory depression and delayed emergence are primarily attributed to residual anesthetic or analgesics [35, 36]. Previous studies have revealed that the use of BIS during general anesthesia could reduce anesthetic consumption and enhance postoperative recovery [37, 38]. As discussed in the previous paragraph, we confirmed that SPI accompanied with hypnosis monitoring can further reduce the time to extubate after anesthesia by reducing intraoperative opioid consumption. In summary, according to the clinical effectiveness of monitoring for hypnosis and pain and the cost-effectiveness of reduced anesthetics and analgesics, SPI accompanied with hypnosis monitoring should be considered an effective monitoring module to facilitate extubation and enhance recovery after anesthesia.

Among the assessments of postoperative outcomes including PONV, pain scores, and analgesic requirement in the PACU, no significant differences were found between the SPI-guided analgesia group and the conventional practice group. The avoidance of high doses of intraoperative opioids should reduce the incidence of PONV [39, 40], and the use of remifentanil is associated with postoperative hyperalgesia [41]. Noteworthy, some included studies routinely administer preventive antiemetics and analgesics in both groups for postoperative complications [11, 19, 20, 25, 26, 29]. These preventive drugs may influence postoperative outcome assessment.

This meta-analysis has several limitations. First, the high risk of bias regarding blinding of opioid administration by anesthesiologists may influence the result of our analysis, which is an inevitable situation in clinical anesthesia practice. Future study designs should avoid performance bias as much as possible. Second, the high heterogeneity of outcomes might be due to the small numbers of included studies and the use of different opioids. Additionally, different surgeries may cause different degrees of pain intensity, which may be one of the important reasons for the high heterogeneity. Third, our meta-analysis included only one study for pediatric surgery and lacked studies of intravenous general anesthesia or sedation procedures. Therefore, whether the conclusions of our study could be applied to children and ambulatory surgery should be confirmed by further studies. Finally, the combined use of regional anesthesia and analgesics other than opioids and the presence/absence of cardiovascular agonists can influence the intraoperative opioid requirement. However, our study did not focus on discussing these factors due to insufficient literature included in the meta-analysis.

## Conclusion

SPI-guided analgesia under general anesthesia was associated with a short extubation time and eye-open time. However, it did not affect the intraoperative opioid requirement. Its effect on ERAS was not correlated with an increased risk of postoperative complication. Notably, based on the subgroup analysis, SPI-guided analgesia with hypnosis monitoring could effectively reduce intraoperative opioid requirement. Thus, it can be a cost effective and reliable option during general anesthesia.
